# Bridging Retinal and Cerebral Neurodegeneration: A Focus on Crosslinks between Alzheimer–Perusini’s Disease and Retinal Dystrophies

**DOI:** 10.3390/biomedicines11123258

**Published:** 2023-12-08

**Authors:** Luigi Donato, Domenico Mordà, Concetta Scimone, Simona Alibrandi, Rosalia D’Angelo, Antonina Sidoti

**Affiliations:** 1 Department of Biomedical and Dental Sciences and Morphofunctional Imaging, Division of Medical Biotechnologies and Preventive Medicine, University of Messina, 98122 Messina, Italy; ldonato@unime.it (L.D.); cscimone@unime.it (C.S.); rdangelo@unime.it (R.D.); asidoti@unime.it (A.S.); 2 Department of Biomolecular Strategies, Genetics, Cutting-Edge Therapies, Euro-Mediterranean Institute of Science and Technology (I.E.ME.S.T.), 90139 Palermo, Italy; domenicomorda@iemest.eu; 3 Department of Veterinary Sciences, University of Messina, 98122 Messina, Italy

**Keywords:** eye, brain, retinal dystrophies, Alzheimer–Perusini, microglia, rhodopsin

## Abstract

In the early stages of Alzheimer–Perusini’s disease (AD), individuals often experience vision-related issues such as color vision impairment, reduced contrast sensitivity, and visual acuity problems. As the disease progresses, there is a connection with glaucoma and age-related macular degeneration (AMD) leading to retinal cell death. The retina’s involvement suggests a link with the hippocampus, where most AD forms start. A thinning of the retinal nerve fiber layer (RNFL) due to the loss of retinal ganglion cells (RGCs) is seen as a potential AD diagnostic marker using electroretinography (ERG) and optical coherence tomography (OCT). Amyloid beta fragments (Aβ), found in the eye’s vitreous and aqueous humor, are also present in the cerebrospinal fluid (CSF) and accumulate in the retina. Aβ is known to cause tau hyperphosphorylation, leading to its buildup in various retinal layers. However, diseases like AD are now seen as mixed proteinopathies, with deposits of the prion protein (PrP) and α-synuclein found in affected brains and retinas. Glial cells, especially microglial cells, play a crucial role in these diseases, maintaining immunoproteostasis. Studies have shown similarities between retinal and brain microglia in terms of transcription factor expression and morphotypes. All these findings constitute a good start to achieving better comprehension of neurodegeneration in both the eye and the brain. New insights will be able to bring the scientific community closer to specific disease-modifying therapies.

## 1. Retinal Degeneration in Alzheimer’s Disease (AD)

Numerous studies have highlighted visual impairments in individuals with AD, often manifesting earlier than other dementia-related symptoms [1,2,3]. Ashok et al. [4] elucidated that these visual changes arise not solely from pathological developments in the visual cortex but also from the loss of Retinal Ganglion Cells (RGC) and degeneration associated with age-related macular degeneration (AMD) [5,6]. Intermediate hard drusen were commonly found in the temporal region of AD eyes compared to older normal eyes. A recent study has shown that, starting from postmortem brain and eye tissues samples from AD donors, there was a significant relationship between cerebral amyloid angiopathy (CAA) level and number of temporal intermediate hard drusen [7]. Other retinal manifestations in AD encompass diminished macular blood flow and astrogliosis [8]. Interestingly, certain eye diseases exhibit hallmark AD histopathological changes, indicating some overlap. For instance, amyloid-beta (Aβ) and iron accumulate in drusen [9,10,11], a defining sign of AMD, while phosphorylated tau (p-tau) and Aβ are found in RGCs, which are primarily affected in glaucoma.

However, in sporadic AD (SAD), which accounts for 85% of AD cases [12], the accumulation of Aβ and p-tau in RGCs is not consistently observed [13,14,15]. Vision-related abnormalities are prevalent in AD [16]. A decrease in the retinal nerve fiber layer (RNFL) thickness, especially in the inferior and superior quadrants [17,18,19,20], due to selective RGC loss, is now recognized as a potential AD diagnostic marker [21,22].

This marker can be non-invasively detected using optical coherence tomography (OCT) and electroretinography (ERG). However, some authors argue that these tests lack the specificity and sensitivity needed for broad clinical applications [19,23,24,25].

The processing of amyloid precursor protein (APP) in the eye, and its impairment in the brain remains a topic of debate, as does the efficiency of Aβ clearance mechanisms in both the brain and the eye. A significant correlation has been observed between Aβ and degeneration in the RGC layer, photoreceptors, and the retinal pigmented epithelium (RPE) [4,26]. Conversely, p-Tau has been identified from the ganglion cell to the outer plexiform layer [27,28]. While the presence of Aβ in drusen is established, the primary factor causing APP metabolism imbalance in RPE cells remains unidentified. Zhao et al. [29] noted that during normal aging, the production and secretion of Aβ1−42 increase in RPE cells. This leads to its deposition at the interface of RPE cells and the outer segments of photoreceptors and in the subretinal space, where it should be cleared by microglia. However, excessive Aβ expression in RPE cells results in AMD-like pathology [30], causing microglia to accumulate Aβ and other cellular debris, leading to inflammation and typical AMD drusen deposition. Rong et al.’s meta-analysis [31] supports the role of Aβ in AMD pathogenesis, highlighting a significant AD–AMD link [32].

Glaucomatous degeneration of RGCs is linked with p-tau, potentially overexpressed due to increased shear stress and other cytoskeletal proteins via the ROCK kinase pathway. AD also affects other eye regions beyond the pars nervosa. The cornea exhibits increased sensitivity [33] and decreased thickness [34,35]. Pupillary abnormalities in AD patients include slower responses to light and target detection tasks [36], exaggerated reactions to dilute tropicamide [37,38], and a reduced baseline size [39]. Additionally, during cognitive tasks, the pupil diameter appears enlarged [40]. Some AD patients develop equatorial supranuclear cataracts [41,42], but the link between lens opacity and AD is debated. While Sun et al. [43] found no optic nerve axonal damage in AD patients, other studies describe axonal degeneration [44,45] and lamina cribrosa thinning [35]. Bayer et al. and Wostyn et al. [46,47] reported elevated intraocular pressure in AD patients. Expectedly, these changes lead to various visual dysfunctions, including impaired color vision, contrast sensitivity, visual acuity, and visual integration, along with visuospatial impairments, reduced macular thickness, visual field loss, and visuomotor deficits [48].

## 2. Histopathological Alterations in Different Eye Cytotypes Due to β-Amyloid Production

**Figure 1:**
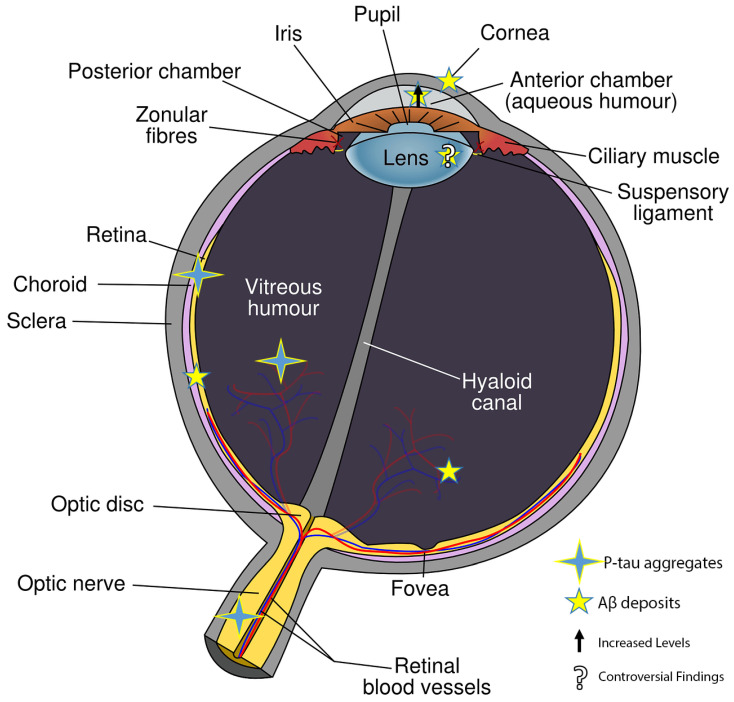
Where Aβ and p-tau were found in eye tissues.

During the progression of AD, multiple eye cell types, both neural and non-neural, exhibit distinct metabolic changes, which subsequently influence gene expression. While these changes have been documented (Figure 1), a comprehensive understanding remains elusive. It is widely believed that a chronic elevation of intraocular pressure (IOP) may instigate the accumulation of not only p-tau in RGCs, but also Aβ, leading to cell death. The processing of APP appears to be consistent in the retina and other eye cell types. This is further supported by the presence of soluble APPα, APPβ, and pathogenic Aβ in the vitreous humor (VH) and aqueous humor (AH), as well as in cerebrospinal fluid (CSF) [49]. Aβ deposits span across all retina layers, from the nerve fiber layer to the photoreceptor layer. Analogous to the brain, retinal Aβ fosters tau protein hyperphosphorylation, leading to its aggregation in structures like neurofibrillary tangles (NFT) [15,50,51]. Elevated IOP has been linked to increased tau oligomerization, resulting in RGC death. The suppression of tau using short interfering RNA (shRNA) has been shown to rescue RGCs, further emphasizing the relationship between p-tau accumulation and cell death [52].

Tau’s role in stabilizing microtubules means that its hyperphosphorylation and aggregation can disrupt anterograde axonal transport, affecting several neuronal functions. For instance, it can hinder mitochondrial transport, leading to energy depletion and the generation of reactive oxygen species (ROS) [53]. In the cornea, both epithelial cells and fibroblasts seem to be involved. Fibroblasts show increased expression of ADAM-10 (α-secretase, the Amyloid Precursor Protein processing enzyme which starts non-amyloidogenic pathway cleaving at residue 697) and BACE-1 (β-secretase, the Amyloid Precursor Protein processing enzyme which starts amyloidogenic pathway cleaving at residue 11) [54], while epithelial cells exhibit heightened APP expression, Aβ accumulation, morphological changes, and an increased rate of apoptosis [34]. Both cell types express APP and produce Aβ [55].

In the retina, notable histopathological markers include a shift towards anaerobic metabolism [56,57], the presence of Aβ plaques [14,58], p-tau deposits [13,52], vascular changes [59,60], and blood–retinal barrier disruptions [61]. Aβ also accumulates in the retinal microvasculature and pericytes [61]. Significant gene expression changes have been observed, such as increased levels of retinal vascular β40 and β42 Aβ fragments and decreased levels of vascular PDGFR-β and LDL-1 [61]. The lens’s involvement in AD is debated; while some researchers have observed increased Aβ aggregation [42,62] and presenilin expression [63], others have not [54,64]. The aqueous humor has shown elevated Aβ levels [65,66], whereas the vitreous humor is rich in AD-associated proteins [49,66]. The choroid appears thinner [67,68], and tau deposition has been observed in the optic nerve [69].

## 3. Retinal Histopathological Abnormalities in AD Mouse Models

Various AD animal models have exhibited retinal Aβ deposits, often accompanied by apoptotic RGCs and axonal degeneration [70,71]. For example, in 3xTG-AD, APP-PS1ΔE9, and APPswe/PS1ΔE9 mouse models, which are known to develop Aβ deposits in the brain, there have been observations of retinal Aβ oligomers and thinning of the retinal nerve fiber layer (RNFL) [6,72,73]. In a glaucoma rat model, the retinal threshold for Aβ1−42 increased in response to elevated intraocular pressure (IOP), aging, and light exposure, leading to RGC apoptosis [52]. Walsh et al. [74] replicated this condition by administering intravitreal injections of Aβ1−42. Their research demonstrated that using agents that either reduce toxic Aβ fragments or induce anti-amyloidogenic mutations can protect RGCs. Lastly, in tauopathy mouse models (where mutations are introduced in MAPT, the tau gene), a direct relationship was established between p-tau, Aβ deposits, and RGC death, highlighting the three primary contributors to the retinal pathology associated with AD [75].

## 4. The Role of the Prion Protein in Retinal Allostasis

In the progression of Alzheimer-Perusini’s disease, the prion protein (PrP) plays a multifaceted role. As described by H. H. Jarosz-Griffiths et al. [76], its function can be likened to the “*Ugly*” character in Ennio Morricone’s renowned film. PrP can act as a conduit for the cytotoxic effects of Amyloid-beta (Aβ), serving as a scavenger receptor. Conversely, it can also block Aβ-derived fragments, preventing their aggregation in the extracellular space, thus acting as a decoy [12]. Currently, two Aβ binding sites on the PrP^C^ structure have been identified: one spanning amino acid residues 95–105 and the other 23–27 [77]. However, only blocking the larger site (residues 95–105) protects neurons from Aβ’s harmful effects. When Aβ oligomers bind to PrP, the downstream tyrosine kinase Fyn pathway is activated, leading to increasingly severe synaptic alterations until their eventual destruction [78].

Like APP, PrP^C^ undergoes various cleavage patterns, termed α-, β-, or γ-cleavage. α-cleavage is facilitated by ADAM17 [79], which belongs to the α-secretase family (also known as TNFα-cleaving enzyme, TACE). This cleavage results in the release of the N-terminal soluble fragment N1 into the extracellular space, while the C-terminal fragment remains attached as C1 via a GPI anchor. Given that C1 lacks the primary Aβ-binding site, this post-translational modification is believed to protect against Aβ-induced toxicity. Conversely, β-cleavage produces a membrane-anchored C-terminal fragment, C2, which contains the entire primary Aβ-binding region, and a released N-terminal fragment N2, which contains the secondary Aβ-binding site spanning residues 23−27. Thus, C2 fragments may facilitate Aβ neurotoxic transmission. γ-cleavage results in a soluble form of PrP^C^. In neurons, the majority (65–80%) of PrP^C^ cleavage occurs at position 111/112, releasing the N1 fragment (α-cleavage). However, in the retina, β-cleavage predominates, primarily due to oxidative stress. This cleavage pattern is believed to protect cells from oxidative damage. The same trend is observed in other eye cell types. This distinction between the eye, especially the retina, and the brain might be attributed to the eye’s constant exposure to light radiation. Another potential factor is the downregulation of ADAM17, the enzyme initiating Aβ’s non-amyloidogenic processing [80]. PrP^C^ itself is believed to regulate ADAM17/TACE levels, indicating a mutual and intricate control mechanism [81,82,83,84,85]. This proteolytic molecule is activated in immune cells, where TNF-α initiates several pathways crucial for their activity [86]. Additionally, PrP^C^ stabilizes the extracellular matrix by interacting with β1 integrin [87,88]. When PrP^C^ expression is reduced, the RhoA-associated coiled-coil-containing kinase (ROCK) pathway is activated. Overactivation of ROCK, through the LIMK-cofilin pathway [89,90], results in a shift in the cytoskeleton status. These changes lead to increased resistance to aqueous outflow, elevated IOP, and ultimately, RGC loss.

In the brain, PrP^C^ acts as a radical scavenger. PrP^C^-knockdown mice exhibit increased susceptibility to intracellular ROS [91]. The N-terminal octapeptide repeat of PrP^C^ coordinates redox-active metals (e.g., Cu, Zn, and Fe) [92,93,94,95], enabling it to act as both a scavenger and a metal transporter [81,96,97]. Given these findings, further research is needed to fully understand PrP’s multifaceted role in the eye [4]. Striebel et al. [32] and others have shown that prion-induced degeneration of photoreceptor cells in mice and humans resembles the pathology of human retinitis pigmentosa caused by retinal protein gene mutations. The Scrapie isoform of the prion protein (PrP^Sc^) [98] has been found to be associated with the base of cilia and swollen cone inner segments. These findings suggest that ciliopathy might be the underlying pathogenic mechanism. PrP^Sc^ has also been detected on the dendrites of cone and rod bipolar cells, extending into ribbon synapses. It is plausible that a similar mechanism might be activated in prion-like diseases, such as AD, as well as in human retinitis pigmentosa (RP) [32].

Not only AD but also other prion and “prion-like” neurodegenerative progressions lead to retinal pathological changes in humans and other species. While there may be some overlap in histopathological abnormalities, each disease seems to have a unique impact on the retina [99]. For instance, in patients with Parkinson’s disease (PD), abnormal α-synuclein oligomers [100] have been observed in the retina, particularly in the nerve fiber (ganglion cell axons), inner plexiform, and ganglion cell body layers [99].

## 5. Different Microglial Phenotypes and Aβ Clearance

Microglia constitute 0.2% of the total retinal cells and 5–10% of the entire CNS. These cells share lineage with monocytes, macrophages, and dendritic cells. They originate from the yolk sac, which is the embryonic location for human hematopoiesis [101], and develop from primitive progenitors [102,103]. Their migration to the CNS occurs post-differentiation [104]. They have been identified in development from embryonic day (E) 8.5–9.5. In mice with compromised circulation (due to sodium-calcium exchanger1, Ncx-1 deficiency), microglia do not reach the brain, suggesting this as their primary migration route [105]. While microglial presence in the human retina is noted by the 10th week of gestation and in mice by E11.5, it is plausible that they enter the brain even earlier [106,107]. This timing is consistent in rats at E12 [108] and in quails at E7 [109].

It is hypothesized that retinal microglial infiltration happens in two sequential migratory waves, based on the spatiotemporal positioning of these cells, both pre- and post-vascularization. Initially, microglia might enter the retina by traversing the vitreal retina surface or migrating from non-neural ciliary regions in the periphery [106,107,110,111]. Later, the invasion likely begins from the optic disc or through blood vessels [112]. Regardless, the arrival of microglia aligns with the differentiation of retinal neurons from retinal progenitor cells (RPCs). A study by Li et al. [104] reported that the outer plexiform layer (OPL) houses 47% of the microglial population, with the remainder primarily in the inner plexiform layer (IPL). The authors speculate that this distribution might mirror the spatiotemporal distribution of synapses in the developing retina. Supporting this theory, synapse formation starts in the nascent IPL around E17, coinciding with the presence of 99% of microglial cells [113,114]. By postnatal day (P)3, approximately 80% remain localized between the developing IPL and the ganglion cell layer (GCL). By P9, microglia are observed in the developing OPL. In adulthood, this pattern persists, with microglia and their processes predominantly found in the inner retina and OPL. In contrast, the outer nuclear layer (ONL) largely lacks them [107,115]. This might be because phagocytosis and general allostasis in this region are overseen by the retinal pigmented epithelium (RPE).

**Figure 2:**
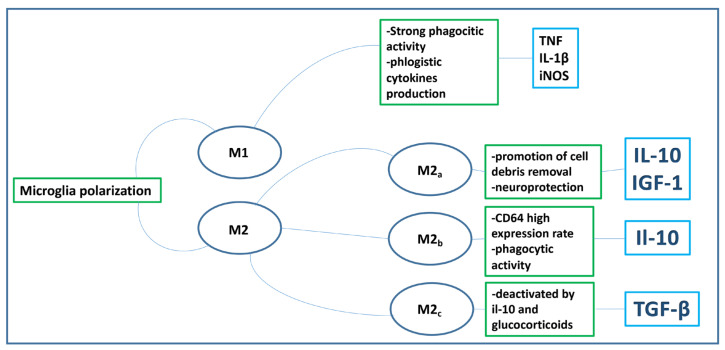
Microglia polarization.

Microglia exhibit a diverse range of phenotypes in response to changes in the CNS microenvironments, including those in the retina. They play a pivotal role in monitoring allostasis within these environments. Historically, two primary microglial phenotypes have been identified. From a dichotomous perspective, microglial activation can be categorized into M1 or M2 phenotypes, similar to macrophages [99]. The M1 phenotype is associated with neuroinflammatory responses, while the M2 phenotype is linked to anti-inflammatory, neuroprotective, and restorative functions. Recent studies have identified at least three subpopulations of the M2 phenotype, labeled M2a-c [116]. Specifically, M2a is involved in the production of IL-10 and IGF-1, facilitating cell debris clearance and neuroprotection [99,116,117]. M2b cells, on the other hand, appear to be activated by inflammatory agents such as IL-1β and LPS and respond by upregulating IL-10 expression [116]. They also exhibit phagocytic activity in AD model brains and have increased levels of CD64, a typical M1 Fc receptor [118]. M2c cells produce significant amounts of TGFβ but are inhibited by IL-10 and glucocorticoids (as illustrated in Figure 2). However, the spectrum of microglial phenotypes is vast. For a more comprehensive overview of microglial phenotypes observed in AD brains, such as damage-associated microglia (DAM), readers are referred to Donato et al. [12].

Our current focus is on specific morphotypes, which are molecular shifts resulting in evident morphological changes. A recent theory has identified two primary microglial states: ramified and amoeboid, with three transitional forms: hyper-ramified, activated, and rod morphotypes [119,120]. Ramified microglia, representing the inactive state, have small, round somas with thin, elongated processes that continuously extend and retract, facilitating their monitoring function [104,119,121]. These cells are distributed in a mosaic pattern [122]. Upon detecting specific microenvironmental stimuli, they transition to the “hyper-ramified” state, characterized by denser, longer, and thicker dendrites and a larger, irregularly shaped soma [121,123]. Persistent harmful stimuli can cause these primed microglia to adopt the “amoeboid” state, which is marked by even larger, more uniformly shaped cell bodies with minimal to no extensions [121,123]. This state is inherently phagocytic, enabling the cells to migrate to inflammation sites and engulf dying neurons and cellular debris [119,124]. “Rod” microglia resemble sausage-shaped cells with few dendrites, sometimes shorter than those of ramified microglia [125,126]. While their exact functions remain elusive, evidence suggests that rod microglia position themselves near neurons, aligning with nerve fibers [125,126,127,128]. Studies have shown that ramified microglia express high levels of P2RY12, associated with surveillance functions, whereas amoeboid microglia predominantly express the phagocytosis marker CD68 [119,129].

Recognizing variations in microglial morphology, especially through retinal neuroimaging, could pave the way for early diagnosis of AD or other neurodegenerative conditions. Fernandez-Arjona et al. [130] defined 15 parameters to facilitate an objective morphometric analysis using automated software (Fraclac, v.2.5; ImageJ v.1.53). During brain development, the amoeboid form of microglia is predominant, especially when synapse remodeling is at its peak. Following this period, within the first two postnatal weeks, they transition to the ramified form [131,132]. A similar transition is observed in the retina. At birth, these immune cells in the retina exhibit an amoeboid structure, extending their dendrites towards the retinal basal side. As the retina matures, they evolve into the ramified morphotype [107]. However, in the event of CNS injury or infection, microglia revert to the amoeboid state [133,134]. Koso et al. highlighted the significance of the zinc finger transcription factor Sall1 in this transition, noting its specific expression in amoeboid retinal microglia. A knockout of this factor can induce a shift from the ramified to the amoeboid form [135]. Both brain and retinal microglia express several common transcription factors [104]. While numerous studies [104,119,127,136,137,138] have identified each morphotype in both the retina and the broader brain, it remains unclear if these morphotypes exhibit identical characteristics across both regions [104]. O’Koren et al. [139] suggested that the cytokine IL-34 might delineate the spatial distribution of distinct microglial subsets in the retina. In healthy conditions, most IL-34- microglial cells are found in the OPL, while IL-34+ cells are predominantly in the IPL. As neurodegeneration progresses, both subsets migrate to the RPE. Interestingly, microglia near damaged retinal neurons show elevated levels of CD11c [140]. In the future, we might harness the potential to reprogram microglial phenotypes to address early pathological developments. Distinct microglial shapes have been observed across various CNS regions, such as the striatum, frontal cortex, and hippocampus, where the soma is larger, and processes are more abundant. Additionally, as one transitions through cerebral cortex layers, the dimensions of microglial cells change [141]. Typically, aging microglia shed their dendrites, resembling a leafless tree, adopting a “dystrophic” appearance [142]. This transformation complicates myelin turnover, further exacerbated by alterations in the myelin itself. Data regarding this phenomenon in the retina is limited, possibly due to its relative lack of this lipid sheath [143].

## 6. Brain–Retina Microglia Axes and Alzheimer–Perusini’s Continuum

Numerous studies [125,136,144,145,146,147,148] have highlighted the association between microglial activation and AD pathology, not only in the brain but also in the retina. The age-induced shift in the epigenetic profile of microglia might influence the onset of specific pathologies, such as AMD or even AMD-like retinal degeneration in AD patients. For instance, Ma et al. [149] observed an age-related upregulation of the AMD-associated gene, C3. Several research groups noted primed microglia in the retinas of 5-week-old 3× transgenic AD mice, suggesting an early microglial response in AD, potentially preceding cerebral involvement [146].

Rod microglia, in particular, warrant special attention due to their potential region-specific involvement in AD [125,136]. In a study by Bachstetter et al. [136], rod microglia were exclusively identified in the parietal cortex of AD patient autopsies, with none found in the hippocampus or temporal cortex when compared to healthy controls. This observation aligns with another study where no significant presence of this microglial morphotype was noted in the hippocampal and cortical brain regions of patients with a history of traumatic brain injury [136]. Furthermore, a correlation between rod microglia and patients with mutated C9orf72 was observed, given its prevalence in this patient group and relative absence in age-matched controls. Interestingly, a higher density of rod microglia in the grey matter of Down’s syndrome AD patients (with three copies of the APP gene) was linked to a more severe pathological progression compared to those with only AD and no chromosomal aberration [125]. Grimaldi et al. [150] examined postmortem AD retinas and identified classic AD histopathological markers, such as Aß plaques and neurofibrillary tangles. Additionally, they reported an increased expression of caspase 3, an AD-associated neurodegeneration marker, and a higher density of iba-1 positive microglia, which also exhibited elevated levels of IL-1ß. However, these microglia maintained normal TREM2 levels, even though AD retinas showed higher TREM2 mRNA levels compared to healthy patients. If these observations are validated by further research, they could serve as effective early diagnostic markers. Understanding the underlying molecular mechanisms could not only facilitate early diagnosis but also pave the way for innovative therapeutic approaches. Moreover, new insights into retinal DAM marker expression, combined with enhanced morphological parameter assessments, could offer a novel diagnostic approach for AD retinopathies [143].

**Figure 3:**
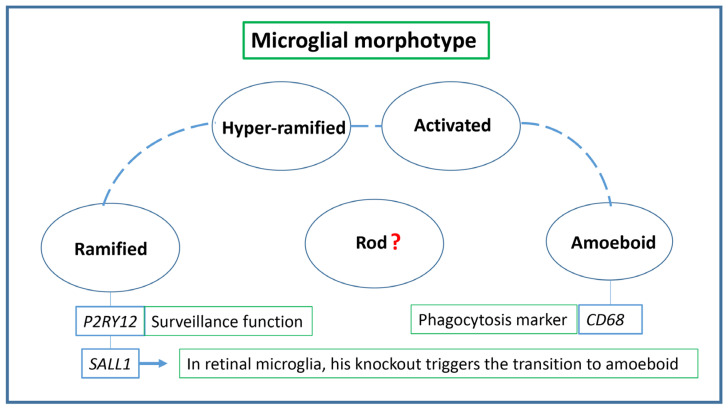
Microglial morphotypes.

In conclusion, another noteworthy microglial phenotype is the perivascular microglia (PM). PM microglia appear to oversee exchanges across the blood–brain barrier (BBB), monitoring compounds entering the CNS from the bloodstream [151]. A study found that drug-induced depletion of PM microglia in AD mice led to the formation of corticovascular amyloid plaques [152]. Figure 3 summarizes the discussed microglial morphotypes.

## 7. The Retina as a Gateway to Early AD Diagnosis

As previously mentioned, AD patients often exhibit visual symptoms related to retinal morphological and functional changes before the manifestation of typical AD symptoms [153]. Many of these changes could serve as early indicators of the disease. For instance, the loss of amacrine and ganglion cells, leading to dysfunction in the RNFL, is frequently reported in the literature and is associated with Aβ oligomerization throughout the retina [75,154]. However, the pattern of Aβ deposition in the retina differs from that in the brain. As discussed in Chiquita et al. [155], advanced studies on neural networks across multiple space–time dimensions [156,157] may provide insights into the parallels between the progression of the disease in the retina and the brain. Additionally, the AD retina exhibits changes related to the immune system (e.g., astrocytes and microglia), vasculature, and electroretinogram (ERG) responses [158]. Events such as cell death and the disrupted allostasis of transition metals (e.g., iron, copper, and zinc) [159] can induce astrocyte and microglia activation [146,160]. This is further complicated by the co-localization with Tau oligomers [161,162] and Aβ plaques. Such a disrupted microenvironment might lead to rhodopsin instability, further exacerbating cell death events [159]. Movio et al. [163] provided a concise summary of the current limitations in analyzing AD in the retina. The data describing the simultaneous structural changes in the retina and AD brain tissue, as reported in the literature, seem inconsistent [155,164]. However, obtaining accurate electrophysiological tracings of RGC in AD remains a challenge, even though it could be pivotal for early diagnosis. In the subsequent sections, we will delve into recent efforts to overcome these challenges.

## 8. Retinal Organoids (RO) and Other Culture Systems

To date, our understanding of neurodegenerative disease-related retinal changes primarily stems from imaging analyses of patients, post-mortem tissue studies, and animal experimental models [165,166,167]. However, a more personalized approach is achievable through the use of induced pluripotent stem cells (hiPSC) and organoid technologies. These can be derived from patients who may be at risk of future neurodegenerative progression, identified either due to genetic predispositions or prolonged exposure to known pollutants, long before symptom onset [163]. Both sporadic AD (SAD) and familial AD (FAD) derived hiPSC cells exhibit similar brain tissue characteristics in vitro [168], suggesting similar characteristics in ROs [169,170]. Key indicators to monitor in these models include rhodopsin stability, neuroinflammatory responses, microvascular changes, and oxidative stress [171]. Given the importance of microglial cells in AD, as discussed previously, a reliable RO model should incorporate these cells, as described in Movio et al. [163]. Considering the significant alterations in AD-associated microglial cells, such as cytokine secretion changes and impaired phagocytosis [172], the use of hiPSC-derived microglial-like cells might be beneficial [173]. Park (2018) proposed a microfluidic system-based approach for an AD 3D model, co-culturing human neurons, astrocytes, and microglia, which effectively maintained astrocyte and microglia activation [159]. Given the significant cell loss observed in the inner retina, a comprehensive 3D organoid model might not be essential for early diagnosis or addressing specific queries [165]. A simpler 2D culture of ganglion and amacrine cells might suffice. However, the inclusion of astrocytes and microglia, combined with microfluidic devices, could enhance the model’s relevance [174]. Utilizing CRISPR/Cas genome editing in hiPSC-derived diseased cells offers the potential to deeply explore the genetic contributions, illuminating the underlying molecular mechanisms [163]. Lastly, when developing neurodegenerative disease models, the intrinsic association with aging should be acknowledged. Strategies such as progerin overexpression, anti-aging gene knockouts, and the induction of reactive oxygen species (ROS) production can make these models more reflective of the actual disease state [175,176].

## 9. Rhodopsin Quantification

Rhodopsin, a combination of opsin and 11-cis-retinal, is a G-protein coupled receptor found in the rod cells of the retina, playing a crucial role in light signal transduction. It serves as a marker for retinal thinning [177,178], which is increasingly recognized as an early indicator of neurodegenerative diseases. Notably, rhodopsin instability and depletion appear to precede the loss of retinal neural cells [159,179]. A study by Ni et al. in 2017 highlighted the role of drosophila rhodopsin receptors as circadian pacemakers in neural cells, suggesting a connection between circadian rhythm disruptions and the etiopathogenesis of neurodegenerative diseases [180], with the discovery of the glymphatic system being particularly noteworthy in this context [181].

Stojanovic et al. proposed a theory in 2004, suggesting that rhodopsin misfolding could be the underlying cause of retinal degeneration observed in conditions like AD and RP [182]. They posited that specific mutations in a high-affinity transmembrane site of rhodopsin, which coordinates with zinc, trigger this misfolding. Interestingly, low zinc levels have been linked to retinal neurodegeneration and night blindness, a result of rod cell death [183]. Elevated zinc concentrations, on the other hand, diminish rhodopsin’s affinity for 11-cis-retinal [184]. The dysregulation of transition metals is closely associated with the onset of neurodegenerative diseases [185].

Recent clinical studies suggest the potential of optical coherent tomography (OCT) as a screening tool for early signs of neurodegenerative progression, given its ability to detect volumetric changes in the retina [186]. As rhodopsin instability is believed to precede cell loss, its quantification could further advance early diagnosis. Liu et al. in 2015 introduced a rhodopsin imaging technique known as nanosecond pulsed scanning laser ophthalmoscopy (SLO), later improved to eSLO (confocal laser ophthalmoscope). This technique offers high-resolution mapping of rhodopsin and pigment epithelium distribution by analyzing lipofuscin autofluorescence in approximately 50 μm resolution pixel clusters. eSLO is designed for compatibility with commonly used clinical imaging instruments [187]. Combining rhodopsin quantification with retinal thickness assessment could pave the way for early diagnosis of brain neurodegeneration [159]. Considering that rhodopsin kinetics slow down with age [188], it is essential to differentiate between pathological and natural reductions in this pigment and establish appropriate benchmarks. While rhodopsin levels are most closely associated with Alzheimer’s disease, they cannot be used for making a differential diagnosis with other neurodegenerative disorders, e.g., Parkinson’s disease. However, when combined with other techniques or early clinical signs, rhodopsin quantification holds significant potential for early diagnosis.

## 10. Conclusions and Future Scenarios

This review offers a comprehensive look at various promising discoveries and theories, some of which are still distant from practical clinical application. To bring these findings to fruition, several challenges must be addressed:

- Enhancing the sensitivity and specificity of certain techniques (as ones mentioned in the previous paragraphs);
- Establishing definitive cut-offs and thresholds (which indicate when a certain accumulation of non-functioning rhodopsin or when a specific modification of the molecule is unequivocally indicative of an emerging neurodegenerative progression);
- Developing a comprehensive diagnostic framework that encompasses various stages and provides refined guidelines for individual evaluations;
- Ensuring global accessibility to advanced equipment and technologies.

The age-old adage suggests that the eyes are windows to the soul. Given their transparency, could the eyes serve as a lens through which early signs of neurodegeneration are detected? Based on our discussions, the potential is promising. However, a deeper understanding of retinal pathophysiology, molecular mechanisms, and gene expression is essential. In many neurodegenerative diseases, specific organs or systems show early signs of affliction. For example, the onset of Parkinson’s disease (PD) is often linked to the olfactory nerve. Such patterns, although unique to each individual, can be grouped into discernible clusters. In the future, advanced bioinformatics tools powered by artificial intelligence (AI) could map these associations on a grand scale, correlating gene expression with structural data. This would bring the scientific community a step closer to ensuring early diagnoses for countless patients globally. Presently, by integrating cutting-edge eye imaging techniques with valuable genetic data, we can potentially screen individuals at medium to high risk of developing dementia. Achieving this goal requires a collaborative effort among neurologists, ophthalmologists, bioinformaticians, and biotechnologists, all working together to elevate scientific knowledge.

## Data Availability

Not applicable.
